# Supplementary material for: Multilayer Optimization for the Quantum Internet
Source: Sci Rep. 2018 Aug 23;8:12690. doi: 10.1038/s41598-018-30957-x (PMC6107661; doi:10.1038/s41598-018-30957-x)
Supplement: Supplementary file 1 — Supplemental Information [file 41598_2018_30957_MOESM1_ESM.pdf]

Supplemental Information

Multilayer Optimization for the Quantum Internet

Laszlo Gyongyosi*[1,2,3] and Sandor Imre[2]

[a] School of Electronics and Computer Science
University of Southampton, Southampton SO17 1BJ, UK;
[b] Department of Networked Systems and Services, Budapest University of Technology and Economics, Budapest, H-1117 Hungary;
[c] MTA-BME Information Systems Research Group, Hungarian Academy of Sciences, H-1051, Budapest, Hungary

*Email: l.gyongyosi@soton.ac.uk

A Appendix

A.1 Notations

The notations of the manuscript are summarized in Table A.1.

Table A.1: Summary of notations.

Notation	*Description*
l	Level of entanglement.
F	Fidelity of entanglement.
N	Entangled quantum network, $N = (V, \mathcal{S})$, where V is a set of nodes, $\mathcal{S}$ is a set of entangled links.
L_l	An l-level entangled link. For an L_l link, the hop-distance is 2^{l-1}.
$d(x,y)_{\mathrm{L}_l}$	Hop-distance of an l-level entangled link between nodes x and y.
$E_{\mathrm{L}_l}(x,y)$	Entangled link $E_{\mathrm{L}_l}(x,y)$ between nodes x and y.
$\lambda_{E_{\mathrm{L}_l}(x,y)}$	Initial entanglement utility of link $E_{\mathrm{L}_l}(x,y)$.
$\lambda'_{E_{\mathrm{L}_l}(x,y)}$	Updated entanglement utility of link $E_{\mathrm{L}_l}(x,y)$.
$B_F(E_{\mathrm{L}_l}(x,y))$	Entanglement throughput of a given L_l-level entangled link $E_{\mathrm{L}_l}(x,y)$ between nodes (x,y).
R_i	An i–th quantum repeater node.
S	A quantum switcher node, switches between entangled connects using its local quantum memory, and applies entanglement swapping.
$\mathcal{G}_m$	Quantum memory utilization graph, directed graph mapped from the network model with abstracted nodes and links.
$\mathcal{G}_{et}$	Entanglement throughput tree is derived from a $\mathcal{G}_m$ quantum memory utilization graph.
ID	Identifier of a node in $\mathcal{G}_{et}$, $ID = \{A, B, \ldots\}$.
S_I	Set of unvisited neighbor nodes of a particular node I.
$\Omega(I,J)$	Cost function between nodes (I,J).
$C(E_{\mathrm{L}_l}(I,J))$	Cost of entangled link $E_{\mathrm{L}_l}(I,J)$.
ζ_J	Cost of quantum storage in node J.

$\Pr(I,J)$	Probability that from node I a node J is selected.
χ, δ	Weighting coefficients in $\Pr(I,J)$.
$\mathcal{M}$	Method of building an $\mathcal{G}_{et}$ entanglement throughput tree.
S'	Set of already reached destination nodes.
$\mathcal{I}$	Set of initial nodes.
$\mathcal{F}_I$	Set of feasible neighboring nodes to node I.
$\mathcal{D}$	Set of destination nodes.
$\alpha_{\mathcal{G}_{et}}$	Entanglement assignment cycle, an optimal assignment (scheduling) of stored entanglement.
$t_s^*(\mathcal{G}_{et})$	Minimal overall storage time at a given $\mathcal{G}_{et}$.
$\mathcal{C}_{\mathcal{G}_{et}}$	Conflict graph of $\mathcal{G}_{et}$. In the $\mathcal{C}_{\mathcal{G}_{et}}$ graph, each vertex corresponds to a directed link of $\mathcal{G}_{et}$ (an entangled connection). There is an edge between two vertices of $\mathcal{C}_{\mathcal{G}_{et}}$, if only the vertices (entangled connections) has a conflict.
$\tau_{n,t}$	Indicator variable, $\tau_{n,t} \in \{0,1\}$, defined as $\tau_{n,t} = \begin{cases} 1, \text{ if } n \text{ is associated at time } t \\ 0, \text{ otherwise.} \end{cases}$
$\wedge(n)$	Set of entangled connects n' that are scheduled in the same time unit t, but the physical link can transmit only n or n'.
$w(n)$	Weight of an entangled link, defined as $w(n) = \begin{cases} 1, & \text{if } F_i = F_{\max} \\ \left\lceil \frac{F_{\max}}{F_i} \right\rceil, & \text{if } F_i < F_{\max} \end{cases},$ where F_i is the fidelity of entangled connection i, $F_{\max}$ is the largest fidelity.
$\mathcal{W}(\mathcal{C}_{\mathcal{G}_{et}})$	Weighted coloring of conflict graph $\mathcal{C}_{\mathcal{G}_{et}}$.
$\Delta(\mathcal{W}(\mathcal{C}_{\mathcal{G}_{et}}))$	Time intervals between each time unit of a given cycle.
$\mathcal{G}_{et}^*$	Optimal entanglement throughput tree.
$t_s(\mathcal{G}_{et}^*)$	Overall storage time at an optimal $\mathcal{G}_{et}^*$.
$B_F(\mathcal{G}_{et}^*)$	Entanglement throughput at an optimal $\mathcal{G}_{et}^*$.
$\lvert\mathcal{P}(\mathcal{G}_{et}^*)\rvert$	Number of entangled links at an optimal $\mathcal{G}_{et}^*$.
$\mathcal{S}_{\mathcal{G}_{et}}^*$	Set of optimal $\mathcal{G}_{et}^*$ entanglement throughput trees.

X	Solution set X with decision variables $\mathrm{X} = \{x_1, \ldots, x_n\}$, where n is the number of all links in a given quantum memory utilization graph $\mathcal{G}_m$, and $x_i \in \{0,1\}$ is defined as $x_i = \begin{cases} 1, \text{ if link } i \text{ of } \mathcal{G}_m \text{ is selected by method } \mathcal{M} \\ 0, \text{ otherwise.} \end{cases}$
$\mathrm{X}_{\mathcal{G}_{et},j} \angle \mathrm{X}_{\mathcal{G}_{et},i}$	Set $\mathrm{X}_{\mathcal{G}_{et},i}$ dominates $\mathrm{X}_{\mathcal{G}_{et},j}$.
κ	Set that contains the best non-dominated solutions that have been found at a particular iteration.
$f_{t_s^*, B_F^*, \lvert\mathcal{P}^*\rvert}(\Theta_i)$	Cost function of classical-layer optimization, where $\Theta_i \in \mathbb{R}^p$ is a p-dimensional real vector of an i-th system state of the quantum network.
$\Theta_i \in \mathbb{R}^p$	A p-dimensional real vector of an i-th system state of the quantum network.
$\Theta_i(j,k,l)$	An i-th system state, where j is the index of a desired optimal system state, k is the index of an optimal system state reproduction step, l is the index of a non-optimal system state event.
T	Total network state, $T(j,k,l) = \{\Theta_i(j,k,l) \mid i = 1, \ldots, S\}$, at a set of S sub-states $\{\Theta_1, \ldots, \Theta_S\}$.
$c(i)$	Number of random system states.
$u(j)$	A unit cost of system change.
C_N	Total cost of classical communication.
A	Distribution-entity of a current system state.
R_{A}	Information transmission rate of A.
ν	Distribution-entity of a system state.
R_ν	Information transmission rate of ν.
$\Theta^{(m)}$	The m-th element of a current network state vector Θ.
$\Theta_i^{(m)}$	The m-th element of Θ_i.
C_e	An environment-dependent cost function.
M	Tuning parameter.
F_{cost}^i	Cost function at a given $\Theta_i(j,k,l)$.
$N_{t_s^*}$, $N_{B_F^*}$, $N_{\lvert\mathcal{P}^*\rvert}$	The number of nodes that require the determination of optimal t_s^*, B_F^* and $\lvert\mathcal{P}^*\rvert$.

$S_{t_s^*}(t)$, $S_{B_F^*}(t)$, $S_{\|\mathcal{P}^*\|}(t)$	The number of classical steps required to find t_s^*, B_F^* and $\|\mathcal{P}^*\|$ at a particular network time t, $t = 1, \ldots, T$.
J	Objective function.
$c^L_{N_{t_s^*}}(t)$, $c^L_{N_{B_F^*}}(t)$, $c^L_{N_{\|\mathcal{P}^*\|}}(t)$	Link cost of classical link L used for the determination of t_s^*, B_F^* and $\|\mathcal{P}^*\|$ at a particular time t.
$\Theta_M(j,k,l)$	Merged system state for the optimization of the classical-layer.
Φ	Merging factor, $\Phi \in [0,1]$.
u	Uniform random number.
x_a, x_b, x_c	Random numbers, $x_a, x_b, x_c \in [0,1]$.
$O_J = f(\psi_{in})$	Output variable, where ψ_{in} is the set of input variables, and $f(\cdot)$ is a function that transfers the uncertainty from the independent input random variables ψ_{in} to the output variable O_J
q	Set of certain variables.
w_i	An input variable under certainty with probability function $\delta_{f_{w_i}}$.
$pc(w_i)$	Probability concentration of w_i.
$\zeta_{i,g}$	Weighting factor.
$O_J^{(i,g)}$	Output variable O_J at a given (i,g).
μ_{w_i}	Mean of w_i.
$w_{i,1}$,$w_{i,2}$	Poth locations of w_i.

Author contributions statement

L.GY. designed the protocol and wrote the manuscript. L.GY. and S.I. analyzed the results. All authors reviewed the manuscript.

Additional Information

Competing Financial Interests: There is no any competing financial interests.
